# Optimizing Patient Engagement with Patient-Reported Outcome Measures Across the Cancer Continuum: A Qualitative Study

**DOI:** 10.1089/pmr.2025.0029

**Published:** 2025-06-05

**Authors:** Eve Seraphina Qing Yi Low, See Mieng Tan, Grace Meijuan Yang, Yu Ke

**Affiliations:** ^1^ Division of Supportive and Palliative Care, National Cancer Centre Singapore, Singapore.; ^2^ Lien Centre for Palliative Care, Duke-NUS Medical School, Singapore, Singapore.; ^3^ Programme in Health Services & Systems Research, Duke-NUS Medical School, Singapore, Singapore.

**Keywords:** implementation, palliative care, patient participation, patient-reported outcomes, oncology, supportive care

## Abstract

**Background:**

Patient-reported outcome measures (PROMs) enhance patient-centered care but routine implementation in oncology settings remains challenging. This study seeks to explore patients’ experiences with routine PROM integration within a health care setting with employed strategies to maximize uptake and inclusivity.

**Methods:**

A qualitative study employing a phenomenological approach was conducted at the National Cancer Centre Singapore. Seven breast cancer patients receiving routine screening using the Distress Thermometer and Problem List (DTPL) as part of a larger supportive care program were purposively sampled. Semi-structured interviews explored how implementation strategies influenced patients’ experiences with PROM usability, accessibility, and perceived impact. Data were analyzed using thematic analysis based on the Consolidated Framework for Implementation Research.

**Results:**

Participants perceived the DTPL as a meaningful PROM that validated their emotions but highlighted that presentation formats greatly influenced perceived simplicity of the tool. While multilingual and hybrid formats improved accessibility, digital literacy and cognitive burden remained as barriers. Education pamphlets provided initial awareness, but sustained engagement was impeded by a lack of time, reminders, and a conducive environment. PROMs were most useful during active treatment when symptoms fluctuated, yet frequent completion led to response fatigue. Timely responses to PROMs reinforced engagement, particularly when linked to referrals or symptom management. Some participants felt that formal PROM reviews by oncologists were unnecessary due to time constraints.

**Conclusion:**

Successful PROM implementation requires balancing simplicity, accessibility, and clinical relevance. Embedding PROMs within broader supportive care programs ensures clinical responsiveness and improves patient outcomes in oncology care.

## Introduction

Patient-reported outcome measures (PROMs) are standardized tools that collect health outcomes directly from patients.^1^ PROM usage in the oncology population is associated with better health-related quality of life, patient satisfaction, symptom management, patient–physician communication, and overall functioning.^2–4^ However, the routine implementation of PROMs in clinical practice remains challenging.^5^ At the patient level, studies have identified multiple barriers pertaining to PROM usability, delayed feedback, and patient engagement.^2,6–8^ Patients may find PROMs burdensome, especially if the tools are not well-integrated into their care experience or if the feedback loop between their reported concerns and clinical actions is weak.^2^ Additionally, differences in health literacy, cultural background, and language proficiency may further hinder PROM completion and perceived relevance.^8^

To address these challenges, various implementation strategies have been proposed in the literature to enhance the uptake and sustainability of PROMs in routine care.^9–11^ In Singapore, a supportive care program employing routine screening using the culturally adapted Distress Thermometer and Problem List (DTPL)^12^ as a PROM for breast cancer outpatients integrates key implementation strategies to maximize patient engagement and inclusivity. First, educational pamphlets were disseminated to improve patient awareness of the screening process, its role in identifying patients in need of additional support, and basic self-management strategies. Second, the PROM tool was culturally adapted for Singapore’s multiethnic and multilingual population, ensuring accessibility through translated tools and a hybrid administration mode. Lastly, structured response care pathways were developed to ensure timely clinical action on distress screening results, enhancing the clinical utility and responsiveness. While previous evaluations^13^ have assessed quantitative metrics such as completion rates and adherence, there is limited insight into how these implementation strategies shape patient engagement with PROMs.

This study aimed to explore patients’ perspectives on the implementation strategies used in a routine PROM screening program for breast cancer patients in Singapore, focusing on their impact on patient engagement, perceived relevance, and usability of the PROM. By identifying key contextual and individual factors that shape PROM uptake, implementation strategies can be further refined to enhance the integration of PROMs into routine oncology care.

## Methods

### Study design and setting

This qualitative study employed a phenomenological approach to explore the experiences of breast cancer patients using the DTPL as a PROM within a supportive care program. The study was conducted at the National Cancer Centre Singapore (NCCS), the largest comprehensive ambulatory cancer center in Singapore, serving ∼65% of adult cancer patients in the public health care sector.^14^ Ethical approval was obtained from the SingHealth Central Institutional Review Board (CIRB 2021-2749).

### Sampling and recruitment

Participants were eligible if they were (1) adults aged 21 years or older, (2) diagnosed with breast or gynecological cancer, (3) receiving routine distress screening via the DTPL as part of the supportive care program during routine outpatient visits with medical oncologists, and (4) able to provide informed consent and participate in an interview in either English or Mandarin. We employed purposive sampling to ensure diversity in cancer stage, time since diagnosis, and ethnicity to capture a broad spectrum of patient experiences.^15^ Recruitment was facilitated by clinical staff within the supportive care program, who directly identified eligible patients for the study team to approach. The recruitment period spanned from January to June 2022. Written informed consent was obtained from all participants before study enrollment.

### Supportive care program employing PROM

**FIG. 1.**
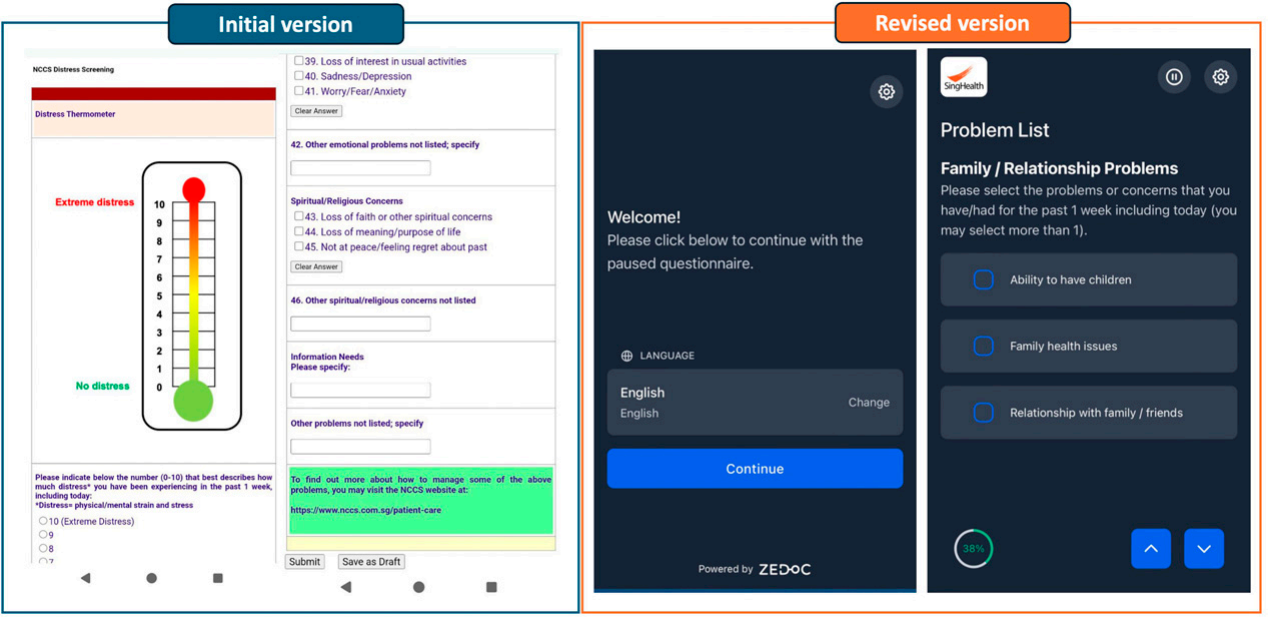
Changes to the web interface of the Distress Thermometer and Problem List. The DTPL web interface was revised from a single-scroll format (left) to a multipage layout with navigation and progress tracking (right) in January 2022.

Since 2019, patients with breast and gynecological cancer at NCCS have received routine screening at each outpatient visit with their medical oncologist using the DTPL.^13^ The original DTPL English version (Supplementary Fig. S1) was culturally adapted from the National Comprehensive Cancer Network^12^ by a multidisciplinary workgroup comprising oncologists, palliative physicians, nurses, pharmacists, and psychologists, before being translated into Singapore’s other official languages (Mandarin, Malay, or Tamil). Patients rate their distress on a 0–10 Likert scale and indicate problems experienced over the past week across five domains: physical, practical, family, emotional, and spiritual. Patients typically complete the DTPL prior to their consultation, either independently via a web-based form or with assistance from clinic staff using paper forms. While the content remained unchanged, the electronic version’s interface was updated in January 2022 to include navigation buttons and progress tracking (Fig. 1). All interviewed participants who completed the DTPL electronically would have experienced this update prior to their interview. Patients scoring a distress score of *≥*6/10 were reviewed by a supportive care nurse.

### Data collection

Semi-structured interviews were conducted via video conferencing. Before each interview, participants completed a baseline survey to collect sociodemographic and clinical information. A trained study team member, with no prior professional relationship with participants, conducted the interviews using a structured guide developed to explore how implementation strategies influenced key aspects of patient engagement. Topics included patient awareness and understanding of the distress screening process; experiences with completing the DTPL, including facilitators and barriers; and perceived usefulness of distress screening in guiding care. Each interview lasted ∼30–45 minutes. Interviews continued until data saturation was achieved, where no new themes emerged from additional sessions.^16^ Given the focused scope of inquiry, thematic convergence was observed early, consistent with previous research suggesting that saturation can be reached with 6–12 interviews.^17^

### Data analysis

Participant characteristics were summarized using descriptive statistics. Interviews were audio-recorded and transcribed verbatim. Qualitative data were analyzed using deductive thematic analysis based on the Consolidated Framework for Implementation Research (CFIR).^18,19^ The CFIR is commonly used to describe facilitators and barriers that affect implementation outcomes and comprises five major domains: innovation (intervention), outer setting (health system), inner setting (outpatient clinics), individuals (implementers, target recipients), and implementation process.

The study team consisted of individuals from diverse disciplinary backgrounds, ensuring a balanced approach to data collection and analysis. Interviews were conducted by a sociology graduate (S.B.O.), while the coding team included a psychology graduate (E.S.Q.Y.L.) and an anthropologist by training (S.M.T.). Both coders are affiliated with the department overseeing the supportive care program. Their contextual familiarity enabled nuanced interpretation while reducing potential biases as neither coder was directly involved in service provision. To enhance analytical rigor, an experienced qualitative researcher with a PhD in pharmacy (Y.K.) served as the adjudicator, resolving coding discrepancies, refining themes, and synthesizing findings in alignment with study objectives.

To ensure transparency and credibility, team discussions were regularly held to critically appraise emergent themes and interpretations. All coders deliberately and continually engage in critical self-evaluation to examine one’s positionality when reviewing the participants’ accounts.^20^ This structured approach to reflexivity ensured analytical integrity while producing findings that authentically reflect participants’ experiences.

## Results

### Participants

A total of seven breast cancer patients were recruited, and their characteristics are summarized in Table 1. The cohort included a diverse representation in terms of age, ethnicity, cancer stage, and employment status, providing a broad spectrum of perspectives.

**Table 1.** Participant Characteristics

Study ID	Age	Years since first cancer diagnosis	Race	Highest education	Employment	Cancer stage	Treatment received
P01	59	24	Chinese	Graduate	Working	4	Immunotherapy
P02	39	1	Chinese	Graduate	Not working	3	Surgery, radiotherapy, chemotherapy, and endocrine therapy
P04	41	6	Filipino	Graduate	Not working	4	Surgery, radiotherapy, and chemotherapy
P05	40	2	Myanmar	Graduate	Working	2	Surgery, radiotherapy, chemotherapy, and endocrine therapy
P07	57	2	Chinese	Graduate	Working	3A	Surgery, radiotherapy, chemotherapy, and endocrine therapy
P08	54	2	Indian	Pre-university	Not working	2	Surgery and chemotherapy
P09	41	2	Chinese	Graduate	Not working	2	Surgery, radiotherapy, chemotherapy, and endocrine therapy

### Themes

Thematic analysis revealed key insights into how implementation strategies influenced patient engagement with PROMs. Table 2 summarizes the key themes and corresponding findings.

**Table 2.** Summary of Key Themes from Interviews

Theme	Key findings
1. PROMs need to be perceived as “simple” enough to be clinically relevant	PROM was generally viewed as effective and easy to complete, offering a means of expressing emotional concerns. Simplicity in format enhanced usability, though some participants desired more open-ended response options.
2. Multilingual and hybrid screening improves accessibility, but capability issues persist	Language options and the flexibility of paper or digital formats supported access. However, digital and health literacy challenges, as well as treatment-related impairments, continued to limit independent use for some.
3. Education provides initial awareness, but sustained PROM engagement requires adequate physical opportunities	Pamphlets and staff explanation were recalled by some, but many noted that clinic overload, emotional distress, and cybersecurity concerns affected their ability to engage meaningfully with the PROM.
4. Timing PROM administration to routine clinical encounters is a double-edged sword	PROMs were seen as most useful during active treatment, when symptom burden was high. However, frequent administration during stable follow-up led to response fatigue and perceived redundancy.
5. Timely clinical responses to PROM results reinforces patient engagement	PROM engagement was strengthened when responses triggered clinical follow-up or supportive care referrals. Conversely, lack of visible follow-up reduced perceived value, especially when trust in clinician communication was already high.

PROM, patient-reported outcome measure.

#### PROMs need to be perceived as “simple” enough to be clinically relevant

Participants described the DTPL as an effective and straightforward PROM that captured their emotional and psychosocial distress. The DTPL further provided an outlet for patients to express concerns that they might otherwise withhold from family members or health care providers. The act of completing the DTPL validated their emotions within a clinical setting.

*“[W]e can express what we are, our feelings there, especially for me [be]cause I cannot- it’s not cannot, I don’t want to let my family know that, what I feel. [Be]cause even before that, I’m not sick, I’m not that person that go and tell, ‘Oh I don’t want this one.’ I just keep to myself like that.”—P04*

*“[N]obody knows the pain we are going thorough. Nobody knows the discomfort… This [screening] system, it calibrates the whole system for us, so we know what are the other concern of my life…It can be distress call, it can be a need to talk. You never know…the support.”—P01*

While the predefined checklist format was praised for its intuitiveness and efficiency in communicating concerns without requiring lengthy explanations, some participants found it restrictive. When their specific concerns did not fit neatly into the predefined options, they suggested incorporating open-ended prompts or free-text fields to allow for elaboration on issues not captured by the structured checklist.

*“I mean you have the question there, then you have the choices there. Then whatever you feel then you just tick.”—P04*

*“Some of the questions that it’s not inside, so I put under others [like]… the medication, I didn’t put it.”—P07*

Additionally, perceived simplicity was influenced by the tool’s presentation format. Initially, the single-scrolling webpage version felt overwhelming to some. A subsequent revision, which introduced multipage navigation, led to mixed reactions. While some participants appreciated the breakdown of questions, others felt that this change added an unnecessary sense of length. Relatedly, visual aids were proposed as a strategy to break down perceived complexity.

*“They updated the format or something… smoother now. Last time it used to- you have to scroll down. But then now, …it’s more like smooth. It’s like a next page thing.”—P02*

*“It’s a bit long. I think 4-page- cannot remember how many page. If can squeeze to two page, it’s best.”—P07*

#### Multilingual and hybrid screening improves accessibility, but capability issues persist

Language accessibility was critical for engagement, with participants emphasizing the importance of completing the DTPL in their preferred language. The availability of both paper and digital formats also provided flexibility. Some participants preferred the digital version for its ability to save responses as drafts, enabling more thoughtful reflections.

*“No need [someone to help], but just got to think through. Sometimes it’s good to have a draft, your draft is good that at least can think… Because if it’s hard paper, I say, I very stressed!”—P07*

Despite these advantages, barriers related to digital and health literacy, particularly among older patients, persisted. Physical limitations due to cancer treatment, such as fatigue, vision impairment, and cognitive difficulties, further posed challenges. Addressing these barriers, participants proposed incorporating audio-guided versions of the PROM to assist patients with literacy or visual impairments.

*“Since I kena this (cancer diagnosis) one already, I [am] a bit forgetful… Because it [ha]s affected into my brain yeah. And my left ear cannot hear.”—P04*

#### Education provides initial awareness, but sustained PROM engagement requires adequate physical opportunities

While participants recalled receiving educational pamphlets and explanations from staff, many did not retain a strong understanding of the purpose of the routine screening. Awareness alone did not lead to consistent PROM completion unless paired with opportunities for engagement, including reminders, time availability, and a conducive environment. Participants described clinic visits as overwhelming, often juggling multiple interactions with different care providers and affecting their capacity to engage.

*“They might have [approached me with hardcopy tool] but if you asked me, I don’t remember… Because there were too many things at that point [in clinic].”—P08*

Emotional readiness also played a role. Some participants felt too emotionally drained from treatment side effects to complete the PROM.

*“Sometimes, you know the emotional is not very good yeah. It’s very, yeah the chemo[therapy] made me into very…very emo[tional].”—P09*

Cybersecurity concerns were a major barrier to physical opportunity. As the PROM was distributed through personalized links via short message service, participants expressed caution about unfamiliar links, especially with rising scam cases. Nevertheless, participants noted that the clear display of trusted health care institution’s name, absence of personal information requests, and prior education in pamphlets helped mitigate these concerns.

*“I started long before that- all those scams came about. So- but now, if someone starting may just feel a bit wary… [But good that] it doesn’t have any kind of personal information that you need to key in, so definitely not, I don’t think it’s that scary.”—P02*

#### Timing PROM administration to routine clinical encounters is a double-edged sword

For some participants, completing the PROM was part of routine follow-up visits, akin to other clinical assessments. Reminders from clinic staff, acknowledgment from oncologists, and an established mindset of compliance helped reinforce this routine.

*“I just answer only. Because they ask me to answer so I just answer. I don’t have special problem.”—P05*

*“I think this is a compulsory, otherwise someone will come to look for you.”—P09*

Many participants expressed that the timing of PROM administration should align with their disease trajectory to maximize its relevance. Many participants felt the PROM was most beneficial during active treatment, when symptom burdens were the highest. At this stage, frequent follow-ups with medical oncologists naturally aligned with more frequent symptom monitoring, ensuring timely intervention for emerging issues,

*“During the cancer treatment right, because all the chemo[therapy] or radiation, there is a lot of effect on our body, especially the chemo[therapy], so like, anxiety and the cannot sleep and the cannot eat, vomit…we cannot stand alone, we need the caregiver. Okay so for that, at the time right, this question would be more suitable.”—P05*

Conversely, frequent administration during closely spaced follow-up visits led to response fatigue. Some participants felt the questions became repetitive, diminishing their motivation to continue engaging with continuously.

*“I think it was a bit repetitive right.? It was like the same thing, but they ask in different ways. So, it’s like, why again you know? I mean, isn’t it the same? Yeah, that did come- crossed my mind.”—P08*

#### Timely clinical responses to PROM results reinforce patient engagement

Participants who received follow-up actions based on their PROM responses, such as referrals to supportive care services or symptom management advice, expressed a stronger sense of engagement. This was particularly evident among those who reported high distress and benefited from additional health care resources provided by the supportive care team.

*“[Nurse name] look at it (DTPL) and she actually respond, more than the others…Consider quite good because initially the anxiety is there…because the- the social worker is so busy they don’t respond.”—P07*

Experiences with oncologists’ use of PROMs were mixed. Some participants appreciated when oncologists referenced their PROM responses to guide clinical decisions, particularly in monitoring and managing treatment side effects. This act reassured them that their PROM responses were acknowledged.

*“At least she (oncologist) is also prepared. At least, you know, she is aware of, you know how I feel, through the survey (PROM). And then she judges on the day itself from the survey (PROM), from what she had, from what I have written in the survey. So, she makes her calls.”—P08*

However, other participants felt that their oncologists already had a strong understanding of their health status, making the formal review of PROMs unnecessary. These participants preferred direct communication with their oncologists, as they trusted them to manage their care effectively. Additionally, participants also recognized that time constraints during consultations limited in-depth discussions.

*“It’s not necessary [for oncologist to review my PROM responses] since she (oncologist) understand [me] very well. She understand the medication history or everything, she understand very well.”—P05*

## Discussion

This study explored patient experiences with routine integration of PROMs in a health care setting where strategies were employed to improve uptake and inclusivity. Key findings extend current knowledge on PROM implementation. First, while a user-friendly interface is important, flexible features tailored to individual preferences are essential. Second, while education is pivotal in initiating PROM usage, ongoing engagement strategies are necessary for sustained completion. Third, structured response pathways can enhance the clinical utility of PROMs, but broader acknowledgment of PROM as part of systemic routine care can engage a larger patient population more effectively. These insights inform future strategies for enhancing PROM implementation in clinical practice.

Our findings reinforce prior literature emphasizing that PROMs must be perceived as simple and easy to complete to maximize uptake.^21–23^ The challenge lies in balancing simplicity with the comprehensiveness required to capture the full range of patient concerns. Patients generally viewed the DTPL as a straightforward PROM, as comprehension issues observed in other studies were likely mitigated by cultural adaptations tailored to the Singapore population.^13,24^ This study extends existing knowledge by illustrating how perceived simplicity is not solely a function of content but also presentation format. Minor digital interface adjustments, such as layout and navigation, could significantly reduce cognitive burden. Human-centered design features,^25^ like adaptive question sequencing and real-time visual progress indicators, can tailor PROMs to individual preferences to promote long-term engagement.

While multilingual and hybrid PROMs improve accessibility, they do not fully accommodate patients with varying literacy levels or cognitive and sensory impairments. To enhance inclusiveness, adaptive multimedia administration should be explored. This transition could be facilitated by the emergence of the multimedia adaptation protocol (MAP), which has successfully transformed PROMs into self-administered multimedia formats across literacy levels.^26,27^ Audio-assisted or verbal PROMs preserve “human touch” through voiceovers while reducing reliance on staff, supporting financial sustainability.^28^ Additionally, flexible digital interfaces are particularly relevant for cancer patients whose symptom burden and cognition fluctuate over time. However, our findings of technological barriers also caution against assuming high smartphone ownership (>90% among adults in Singapore) translates to functional digital literacy.^29,30^ Older adults often use mobile devices for leisure rather than functional tasks, presenting a barrier to digital PROM adoption. Effective PROM integration requires broader societal efforts to promote digital literacy and technology adoption in health care. Strengthening PROM implementation must align with evolving patient behaviors and health care strategies to ensure long-term assimilation into routine care.

Our results further provide new insights into the interplay between PROM education, awareness, and engagement. While passive strategies like pamphlets raised initial awareness, sustained engagement required direct clinician interaction and reinforcement. One major determinant bridging awareness to action was the extent to which patients internalized the value of self-reporting health outcomes. Patients must understand how PROM responses directly inform their care, reducing uncertainty about the clinical impact of screening—one of the most persistent barriers to PROM uptake.^31–34^ Beyond active messaging, automated reminders and data privacy assurances are essential for fostering trust, particularly amid growing digital security concerns.^35^ To fully integrate PROMs into routine care, strategies must address both digital trust and patient motivation, ensuring PROMs are perceived as meaningful tools for improving health outcomes.

With no universal standard for the optimal frequency and timing of routine PROM administration, our study explored screening at each outpatient visit.^36,37^ This approach enabled more frequent monitoring during treatment periods of heightened symptom variability and unexpectedly helped normalize PROM use as part of routine care. However, timing alone did not drive engagement as patients emphasized the need for the right conditions, environment, and emotional readiness. Aligning PROMs with clinical encounters enhances clinical relevance but must be complemented by strategies that reduce patient burden. Offering flexible options of post-visit completion or symptom-triggered assessments can improve engagement without adding unnecessary stress.

Patients recognized the value of timely clinical feedback on PROM responses, aligning with evidence that PROMs enhance treatment monitoring, individualized care, and overall satisfaction.^38–40^ Notwithstanding, our findings suggest that oncologist engagement alone is not the primary driver of PROM uptake. While patients appreciated oncologists responding to their PROM results, they did not view such immediate responses as essential, acknowledging time constraints and competing clinical priorities within their consultation slot. This perspective shifts PROMs from a patient–provider tool to an institution-wide intervention, particularly when embedded in broader supportive care programs like in our study. Positioning PROMs as a mechanism for systematic access to allied health and nursing support reinforces its role as a safety net, facilitating timely care without relying solely on oncologists to sustain patient engagement.

Several limitations should be noted. First, conducting this study in a single national cancer center provided valuable context-specific insights but may not reflect variations in PROM integration across different health care settings, including the public–private divide. Second, self-selection bias may have influenced findings, as participants who agreed to be interviewed likely had stronger opinions about PROMs. Third, while the sample size was small, purposive sampling yielded rich and detailed narratives. Although the findings may not be broadly generalizable, they offer transferable insights to settings with similar supportive care structures and universal health coverage. Future research should explore multisite implementations and engage patients with poor PROM uptake to expand understanding of engagement barriers.

## Conclusion

This study underscores the critical role of implementation strategies in optimizing PROM integration in oncology care. Effective integration requires adaptable presentation formats, digital literacy support, and institutional commitment. While multilingual and hybrid administration improve accessibility, adaptive multimedia approaches are needed for inclusivity. Aligning PROMs with clinical encounters enhances relevance but must allow flexibility for patient readiness. Embedding PROMs within broader supportive care frameworks, rather than relying solely on oncologists, maximizes their impact. To sustain long-term uptake, PROM implementation must evolve alongside digital literacy efforts and patient-centered strategies that reinforce its clinical value.

## Abbreviations Used

CFIR: Consolidated Framework for Implementation Research
DTPL: Distress Thermometer and Problem List
MAP: multimedia adaptation protocol
NCCS: National Cancer Centre Singapore
PROM: patient-reported outcome measure
