# Supplementary material for: Optimizing Patient Engagement with Patient-Reported Outcome Measures Across the Cancer Continuum: A Qualitative Study
Source: Palliat Med Rep. 2025 Jun 5;6(1):333–41. doi: 10.1089/pmr.2025.0029 (PMC12410332; doi:10.1089/pmr.2025.0029)
Supplement: Supplementary Figure S1 [file pmr.2025.0029_supplementary_figure_s1.docx]

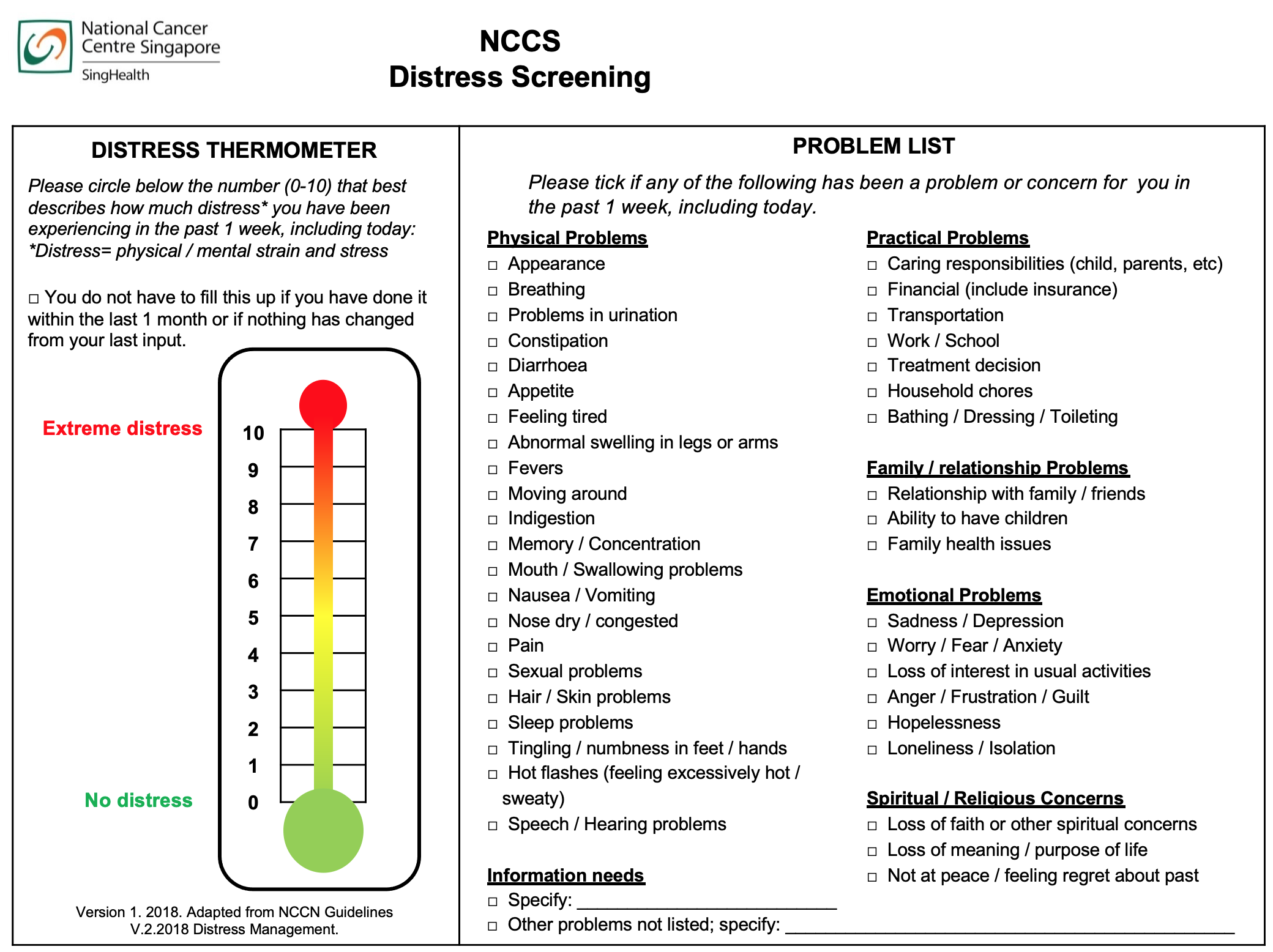


**Supplementary Figure 1.** Distress Thermometer and Problem List adapted from the National Comprehensive Cancer Network.
